# Supplementary material for: Polygrammar: Grammar for Digital Polymer Representation and Generation
Source: Adv Sci (Weinh). 2022 Jun 9;9(23):2101864. doi: 10.1002/advs.202101864 (PMC9376847; doi:10.1002/advs.202101864)
Supplement: Supplementary file 1 — Supporting Information [file ADVS-9-2101864-s001.pdf]

ADVANCED SCIENCE
Open Access

Supporting Information

for *Adv. Sci.*, DOI 10.1002/advs.202101864

Polygrammar: Grammar for Digital Polymer Representation and Generation

*Minghao Guo, Wan Shou, Liane Makatura, Timothy Erps, Michael Foshey and Wojciech Matusik**

ADVANCED SCIENCE
Open Access

Supporting Information

for *Adv. Sci.*, DOI: 10.1002/advs.202101864

Polygrammar: Grammar for Digital Polymer Representation and Generation

*Minghao Guo, Wan Shou, Liane Makatura, Timothy Erps, Michael Foshey, Wojciech Matusik**

Supporting Information

Polygrammar: Grammar for Digital Polymer Representation and Generation

*Minghao Guo, Wan Shou, Liane Makatura, Timothy Erps, Michael Foshey, Wojciech Matusik**

S1. Production Rules of Global Controllable Grammar

The basic idea of global controllable grammar is to use a message to collect global information about the chain. The message passes back and forth between the left and right ends of the string. It is achieved by swapping the message's position with the adjacent symbol's one at a time. This swapping continues along a certain direction until the message gets to the string end. At each position swap, the message updates its parameters to collect the information required for the control setting. The full set of the production rules for the message passing mechanism is illustrated as follows. Note that in this case, all of the symbols are non-terminal symbols.

p_1 :	$None$	$<\mathcal{X}>$	$None$:	$None$	$\rightarrow h\mathcal{M}(1,1,0,1)\mathcal{H}h$
p_2 :	$None$	$<\mathcal{X}>$	$None$:	$None$	$\rightarrow h\mathcal{H}\mathcal{M}(1,1,0,0)h$
p_3 :	$None$	$<\mathcal{X}>$	$None$:	$None$	$\rightarrow s\mathcal{M}(1,0,1,1)\mathcal{S}s$
p_4 :	$None$	$<\mathcal{X}>$	$None$:	$None$	$\rightarrow s\mathcal{S}\mathcal{M}(1,0,1,0)s$
p_5 :	$None$	$<lower>$	$\mathcal{M}(l,r,t,d)$:	$d==1$ and $l<L$ and $r<R$	$\rightarrow h\mathcal{M}(0,0,0,0)\mathcal{H}$
p_6 :	$None$	$<lower>$	$\mathcal{M}(l,r,t,d)$:	$d==1$ and $l<L$	$\rightarrow s\mathcal{M}(0,0,1,0)\mathcal{S}$
p_7 :	$lower$	$<\mathcal{M}(l,r,t,d)>$	$upper$:	$d==1$	$\rightarrow Null$
p_8 :	$\mathcal{M}(l,r,t,d)$	$<\mathcal{H}>$	$\mathcal{H}$:	$d==0$	$\rightarrow \mathcal{M}\left(l+1,\frac{r*l+1}{l+1},0,d\right)$
p_9 :	$\mathcal{M}(l,r,t,d)$	$<\mathcal{H}>$	h:	$d==0$	$\rightarrow \mathcal{M}\left(l+1,\frac{r*l+1}{l+1},0,d\right)$
p_{10} :	$\mathcal{M}(l,r,t,d)$	$<\mathcal{H}>$	$\mathcal{S}$:	$d==0$	$\rightarrow \mathcal{M}\left(l+1,\frac{r*l+1}{l+1},1,d\right)$
p_{11} :	$\mathcal{M}(l,r,t,d)$	$<\mathcal{S}>$	$\mathcal{H}$:	$d==0$	$\rightarrow \mathcal{M}\left(l+1,\frac{r*l}{l+1},0,d\right)$
p_{12} :	$\mathcal{M}(l,r,t,d)$	$<\mathcal{S}>$	$\mathcal{S}$:	$d==0$	$\rightarrow \mathcal{M}\left(l+1,\frac{r*l}{l+1},1,d\right)$
p_{13} :	$\mathcal{M}(l,r,t,d)$	$<\mathcal{S}>$	s:	$d==0$	$\rightarrow \mathcal{M}\left(l+1,\frac{r*l}{l+1},1,d\right)$
p_{14} :	$lower$	$<\mathcal{M}(l,r,t,d)>$	$upper$:	$d==0$ and $t==1$	$\rightarrow \mathcal{S}$
p_{15} :	$lower$	$<\mathcal{M}(l,r,t,d)>$	$upper$:	$d==0$ and $t==1$	$\rightarrow \mathcal{H}$
p_{16} :	$upper$	$<\mathcal{M}(l,r,t,d)>$	$upper$:	$t==1$	$\rightarrow \mathcal{S}$
p_{17} :	$upper$	$<\mathcal{M}(l,r,t,d)>$	$upper$:	$t==0$	$\rightarrow \mathcal{H}$

$p_{18}: \mathcal{M}(l,r,t,d)\ <lower>\quad None:\quad d==1\ and\ l<L\ and\ r<R \rightarrow \mathcal{HM}(0,0,0,1)h$

$p_{19}: \mathcal{M}(l,r,t,d)\ <lower>\quad None:\quad d==1\ and\ l<L \rightarrow \mathcal{SM}(0,0,1,1)s$

$p_{20}:\ upper\ <\mathcal{M}(l,r,t,d)>\quad lower:\quad d==0 \rightarrow Null$

$p_{21}:\ \mathcal{H}\quad <\mathcal{H}>\quad \mathcal{M}(l,r,t,d):\quad d==1 \rightarrow \mathcal{M}\left(l+1,\frac{r*l+1}{l+1},0,d\right)$

$p_{22}:\ h\quad <\mathcal{H}>\quad \mathcal{M}(l,r,t,d):\quad d==1 \rightarrow \mathcal{M}\left(l+1,\frac{r*l+1}{l+1},0,d\right)$

$p_{23}:\ \mathcal{S}\quad <\mathcal{H}>\quad \mathcal{M}(l,r,t,d):\quad d==1 \rightarrow \mathcal{M}\left(l+1,\frac{r*l+1}{l+1},1,d\right)$

$p_{24}:\ \mathcal{H}\quad <\mathcal{H}>\quad \mathcal{M}(l,r,t,d):\quad d==1 \rightarrow \mathcal{M}\left(l+1,\frac{r*l}{l+1},0,d\right)$

$p_{25}:\ \mathcal{S}\quad <\mathcal{H}>\quad \mathcal{M}(l,r,t,d):\quad d==1 \rightarrow \mathcal{M}\left(l+1,\frac{r*l}{l+1},1,d\right)$

$p_{26}:\ s\quad <\mathcal{H}>\quad \mathcal{M}(l,r,t,d):\quad d==1 \rightarrow \mathcal{M}\left(l+1,\frac{r*l}{l+1},1,d\right)$

$p_{27}:\ upper\ <\mathcal{M}(l,r,t,d)>\quad lower:\quad d==1\ and\ t==1 \rightarrow \mathcal{S}$

$p_{28}:\ upper\ <\mathcal{M}(l,r,t,d)>\quad lower:\quad d==1\ and\ t==0 \rightarrow \mathcal{H}$

Similar to the PolyGrammar in the main paper, the global controllable grammar is also a context-sensitive parametric grammar. $\mathcal{M}$ denotes the message, $lower$ indicates the lower case symbols, containing h and s, $upper$ indicates the upper case symbols, containing $\mathcal{H}$ and $\mathcal{S}$. For each production rule in the grammar, the "→" separates the predecessor and the successor. The symbol to be replaced is inside the "< >". The contexts are the symbols located at both sides of "< >" in the predecessor ($None$ indicates no constraints). Parameters of message symbol $\mathcal{M}$ locates inside "( )". The logic condition of the parameters for each production rule is between ":" and "→" ($None$ also indicates no constraints here). During the production process, the production rule can only be applied to a symbol when both its context and the logic condition are satisfied. The production process will stop when no production rules can be invoked, i.e. , for each symbol of the string, there are no production rules that can meet the condition and the contexts of the symbol.

With this set of production rules, we can control two constraints of the polyurethane chain: the chain length L and the ratio R of hard segment to soft segment. The message symbol $\mathcal{M}$ propagates back and forth between the left and right end of the string, collects global information of the chain, and determines how to expand the string meeting the constraints.

The propagation is achieved by switching the message's position with the adjacent symbol's along a certain direction one at a time until the message gets to the string end. At each swap, the message updates its parameters to collect information needed for the control setting. There are four parameters in $\mathcal{M}$ in total: l indicates the current chain length, r indicates the current chain's ratio of hard segment to soft segment, t is an auxiliary parameter to record the symbol that $\mathcal{M}$ is switching with (0 for $\mathcal{H}$, 1 for $\mathcal{S}$), and d indicates the direction that the message is propagating (0 for right, 1 for left). As for the roles that each production rule serves, p_1, p_2, p_3, and p_4 initialize the start symbol $\mathcal{X}$. Taking p_5, p_6, and p_7 together, when the left-propagating message arrives at the left end, the string is expanded according to the collected information and the constraints. Meanwhile, this left-propagating message disappears, and a right-propagating message is generated. This right-propagating message continually switches its position with its right-neighbor symbol using the rules p_8, p_9, p_{10}, p_{11}, p_{12}, p_{13}, p_{14}, p_{15}, p_{16}, and p_{17}. Similarly, p_{18}, p_{19}, and p_{20} expand the string when the right-propagating message arrives at the right end and generate a left-propagating message. p_{21}, p_{22}, p_{23}, p_{24}, p_{25}, p_{26}, p_{27}, p_{28}, p_{16}, and p_{17} propagate the left-propagating message by iteratively switching its position with its left-neighbor symbol. A detailed illustrative example of the message passing mechanism is shown in Figure. S1.

The example in Figure. S1 illustrates the production process under the constraints $L = 3$, $R = 0.67$, where the chain length is 3 and the ratio of hard segment to soft segment is lower than 0.7. At step 1, p_1 expands the initial symbol $\mathcal{X}$. The parameters of $\mathcal{M}$ indicate that the message is currently propagating along the left direction ($d = 1$). Since it has already reached the left end of the string and the current ratio $r = 1$ is larger than the constraint $R = 0.67$, at step 2, p_6 expands the left end of the string with $\mathcal{S}$ and also generates a new message $\mathcal{M}$ ($l = 0$, $r = 0$) propagating along the right direction ($d = 0$). Meanwhile, the previous message disappears by p_7. Then at step 3 and step 4, the message propagates along the right direction by

switching position with its right neighbor. The switching is assisted with the auxiliary parameter t. Also during the propagation, l and r are updated to record the information of chain length and the ratio of hard segment to soft segment. At step 5, the message reaches the right end of the string, so p_{18} expands the string with $\mathcal{H}$ as the ratio $r = 0.5$ is smaller than $R = 0.67$. A new left-propagating message is generated and the old message disappears. At step 6, 7, and 8, the message carries on the propagation and collects the information. At step 9, when the message reaches the left end, since the chain length $l = 3$ meets the constraints $L = 3$, the message disappears and the production process concludes.

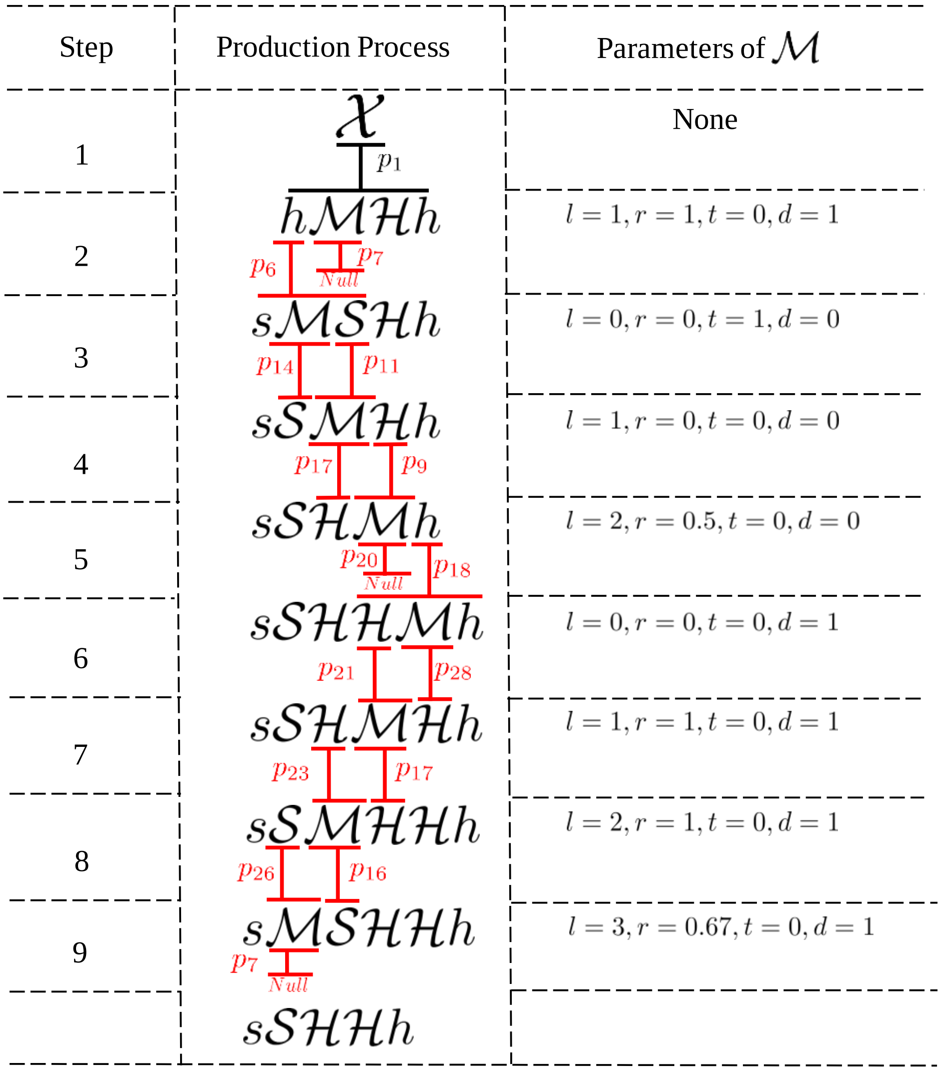

Figure. S1. An illustrative example of the message passing mechanism with constraints $L = 3$, $R = 0.67$.

S2. Collected Dataset of Polyurethane from Literature

Name	BigSMILES
Diisocynates	
TDI	CC1=CC=C(NC(=O)>)C=C1NC(=O)>
MDI	>C(=O)Nc1ccc(Cc2cccNC(=O)>cc2)cc1
HDI	>C(=O)NCCCCCCNC(=O)>
IPDI	CC1(C)CC(NC(=O)>)CC(CNC(=O)>)(C)C1
DBDI	>C(=O)Nc1ccc(CCc2ccc(NC(=O)>)cc2)cc1
HMDI	>C(=O)NC1CCC(CC2CCC(NC(=O)>)CC2)CC1
NDI	>C(=O)Nc1cccc2c(NC(=O)>)cccc12
TMDI	CC(CCNC(=O)>)CC(C)(C)CNC(=O)>
Polyols	
PTMO/ PTHF	<OCCC{[<]OCCCC[>]}O<
PEG/ PEO	<OCC{[<]OCC[>]}O<
PEA	<OCCOC(=O)CCCCC(=O){[<]OCCOC(=O)CCCCC(=O)[>]}O<
PBA	<OCCCCOC(=O)CCCCC(=O){[<]OCCCCOC(=O)CCCCC(=O)[>]}O<
PBU	CC=CC
PCL/ PCD	<OCCCCCC(=O)OCCCCCC(=O){[<]OCCCCCC(=O)OCCCCCC(=O)[>]}O<
PHA	<OCCCCCCOC(=O)OCCCCCCOC(=O)O{[<]OCCCCCCOC(=O)OCCCCCCOC(=O)O[>]}O<
PET	<OC(=O)c1ccc(cc1)C(=O)OCC{[<]OC(=O)c1ccc(cc1)C(=O)OCC[>]}O<
PLA	<OC(C)C(=O){[<]OC(C)C(=O)[>]}O<
CHDM	<OCC1CCC(CC1)C{[<]OCC1CCC(CC1)C[>]}O<
Poly bd	<OCC=CCCC(C=C)CC=CC{[<]OCC=CCCC(C=C)CC=CC}O<
Chain Extenders	
BDO/ BD/BG	<OCCCCO<

EG	<OCCO<
DEG	<OCCOCCO<
DAPO	<Nc1ccc(cc1)-c2nnc(o2)-c3ccc(cc3)N<
DAB	<Nc1ccc(CCc2ccc(N<)cc2)cc1
DAPy	C1=CC(=NC(=C1)N(<))N<
MDA	<Nc1ccc(Cc2ccc(N<)cc2)cc1

S3. Examples of Translation from SMILES

1. Input SMILES:

   CC1(C)CC(NC(=O)OCCCCOCCCCOCCCCOCCCCOC(=O)NCC2(C)CC(NC(=O)
   OCCCCOC(=O)NCC3(C)CC(NC(=O)OCCCCOC(=O)NCC4(C)CC(NC(=O)OCC
   CCOCCCCOC(=O)NCC5(C)CC(NC(=O)OCCCCOC(=O)NCC6(C)CC(NC(=O)O
   CCCCOCCCCOCCCCOCCCCOCCCCO)CC(C)(C)C6)CC(C)(C)C5)CC(C)(C)C
   4)CC(C)(C)C3)CC(C)(C)C2)CC(C)(CN=C=O)C1

   Translation Results:

   Component types: IPDI, PTMO, BDO

   Symbolic hypergraph string: $\mathcal{HSHHHSHHS}$

   Production rules: $\{p_1, p_{10}, p_{12}, p_9, p_9, p_{10}, p_{12}, p_9, p_{10}, p_5, p_{14}\}$

2. Input SMILES:

   Cc1ccc(NC(=O)OCCCCOC(=O)Nc2cc(NC(=O)OCCCCOCCCCOCCCCOCCCCO
   CCCCOCCCCOCCCCOC(=O)Nc3cc(NC(=O)OCCOC(=O)Nc4cc(NC(=O)OCCO
   C(=O)Nc5cc(NC(=O)OCCCCOC(=O)Nc6cc(NC(=O)OCCCCO)ccc6C)ccc5
   C)ccc4C)ccc3C)ccc2C)cc1NC(=O)OCCCCO

   Translation Results:

   Component types: TDI, PTMO, EG

Symbolic hypergraph string: $\mathcal{SHSHHHSHSHS}$

Production rules: $\{p_1, p_4, p_{10}, p_6, p_4, p_6, p_3, p_3, p_4, p_6, p_4, p_8, p_{14}\}$

3. Input SMILES:

CC(OC(=O)Nc1cccc2c(NC(=O)OCCCCOC(=O)Nc3cccc4c(NC(=O)OCCCCOC(=O)Nc5cccc6c(NC(=O)OC(=O)C(C)OC(=O)C(C)OC(=O)Nc7cccc8c(NC(=O)OC(=O)C(C)OC(=O)Nc9cccc%10c(NC(=O)OCCCCOC(=O)Nc%11cccc%12c(N=C=O)cccc%11%12)cccc9%10)cccc78)cccc56)cccc34)cccc12)C(=O)OC(=O)Nc1cccc2c(NC(=O)OCCCCOC(=O)Nc3cccc4c(NC(=O)OC(C)C(=O)OC(=O)Nc5cccc6c(NC(=O)OCCCCOC(=O)Nc7cccc8c(NC(=O)OCCCCOC(=O)Nc9cccc%10c(NC(=O)OCCCCOC(=O)Nc%11cccc%12c(N=C=O)cccc%11%12)cccc9%10)cccc78)cccc56)cccc34)cccc12

Translation Results:

Component types: NDI, PLA, BDO

Symbolic hypergraph string: $\mathcal{HHSHSHHHSHHSHHHH}$

Production rules: $\{p_2, p_6, p_{12}, p_3, p_9, p_3, p_{10}, p_4, p_{12}, p_6, p_9, p_4, p_9, p_6, p_9, p_3, p_5, p_{11}\}$

4. Input SMILES:

O=C(NC1CCC(CC2CCC(NC(=O)OCCOCCOC(=O)NC3CCC(CC4CCC(NC(=O)OCCOC(=O)NC5CCC(CC6CCC(NC(=O)OCCOC(=O)NC7CCC(CC8CCC(NC(=O)OCCOCCOC(=O)NC9CCC(CC%10CCC(NC(=O)OCCOCCOC(=O)NC%11CCC(CC%12CCC(NC(=O)OCCOCCOC(=O)NC%13CCC(CC%14CCC(NC(=O)OCCOC(=O)NC%15CCC(CC%16CCC(NC(=O)OCCOCCO)CC%16)CC%15)CC%14)CC%13)CC%12)CC%11)CC%10)CC9)CC8)CC7)CC6)CC5)CC4)CC3)CC2)CC1)OCCOCCOCCOCCO

Translation Results:

Component types: HMDI, PEG, DEG

Symbolic hypergraph string: $\mathcal{SHHHHHHHHHHHS}$

Production rules: $\{p_1, p_{10}, p_3, p_3, p_3, p_3, p_3, p_3, p_3, p_3, p_3, p_3, p_4, p_8, p_{14}\}$

5. Input SMILES:

```
O=C=Nc1ccc(Cc2ccc(NC(=O)OCCCCOCCCCOCCCCOC(=O)Nc3ccc(Cc4cc
c(NC(=O)OCCOCCOC(=O)Nc5ccc(Cc6ccc(NC(=O)OCCCCOC(=O)Nc7ccc
(Cc8ccc(NC(=O)OCCOCCOC(=O)Nc9ccc(Cc%10ccc(NC(=O)OCCCCOCCC
COCCCCOCCCCOCCCCOC(=O)Nc%11ccc(Cc%12ccc(NC(=O)OCCCCOC(=O)
Nc%13ccc(Cc%14ccc(N=C=O)cc%14)cc%13)cc%12)cc%11)cc%10)cc9
)cc8)cc7)cc6)cc5)cc4)cc3)cc2)cc1
```

Translation Results:

Component types: MDI, PTMO, DEG

Symbolic hypergraph string: $\mathcal{HSHHSHHSHSH}$

Production rules: $\{p_1, p_{10}, p_{12}, p_9, p_{10}, p_{12}, p_9, p_{10}, p_{12}, p_{10}, p_{12}, p_5, p_{11}\}$

6. Input SMILES:

```
O=C=NCCCCCCNC(=O)OCCCCCC(=O)OCCCCCC(=O)OCCCCCC(=O)OCCCCCC
(=O)OCCCCCC(=O)OCCCCCC(=O)OCCCCCC(=O)OCCCCCC(=O)OCCCCCC(=
O)OCCCCCC(=O)OCCCCCC(=O)OCCCCCC(=O)OC(=O)NCCCCCCNC(=O)OCC
OC(=O)NCCCCCCNC(=O)OCCOC(=O)NCCCCCCNC(=O)OCCCCCC(=O)OCCCC
CC(=O)OCCCCCC(=O)OCCCCCC(=O)OCCCCCC(=O)OCCCCCC(=O)OC(=O)N
CCCCCCNC(=O)OCCCCCC(=O)OCCCCCC(=O)OC(=O)NCCCCCCNC(=O)OCCO
C(=O)NCCCCCCN=C=O
```

Translation Results:

Component types: HDI, PCL, EG

Symbolic hypergraph string: $\mathcal{HSHHHSHSHH}$

Production rules: $\{p_1, p_{10}, p_{12}, p_9, p_9, p_{10}, p_{12}, p_{10}, p_{12}, p_9, p_5, p_{11}\}$

7. Input SMILES:

```
CC(O)C(=O)OC(C)C(=O)OC(=O)Nc1ccc(Cc2ccc(NC(=O)OC(C)C(=O)O
C(=O)Nc3ccc(Cc4ccc(NC(=O)OCCCCOC(=O)Nc5ccc(Cc6ccc(NC(=O)O
CCCCOC(=O)Nc7ccc(Cc8ccc(NC(=O)OC(C)C(=O)OC(C)C(=O)OC(C)C(
=O)OC(=O)Nc9ccc(Cc%10ccc(NC(=O)OC(C)C(=O)OC(=O)Nc%11ccc(C
c%12ccc(NC(=O)OC(C)C(=O)OC(=O)Nc%13ccc(Cc%14ccc(NC(=O)OCC
CCOC(=O)Nc%15ccc(Cc%16ccc(NC(=O)OC(C)C(=O)O)cc%16)cc%15)c
c%14)cc%13)cc%12)cc%11)cc%10)cc9)cc8)cc7)cc6)cc5)cc4)cc3)
cc2)cc1
```

Translation Results:

Component types: MDI, PLA, BDO

Symbolic hypergraph string: $\mathcal{SHSHHHSHSHSHHS}$

Production rules: $\{p_2, p_{12}, p_{10}, p_{12}, p_9, p_9, p_{10}, p_{12}, p_{10}, p_{12}, p_{10}, p_{12}, p_9, p_{10}, p_8, p_{14}\}$

8. Input SMILES:

```
C=CC(CC=CO)CCC=CCOC=CCC(C=C)CCC=CCOC=CCC(C=C)CCC=CCOC=CCC
(C=C)CCC=CCOC(=O)NC1CC(C)(C)CC(C)(CNC(=O)OC=CCC(C=C)CCC=C
COC(=O)NC2CC(C)(C)CC(C)(CNC(=O)Nc3cccc(NC(=O)NC4CC(C)(C)C
C(C)(CNC(=O)OC=CCC(C=C)CCC=CCOC=CCC(C=C)CCC=CCOC=CCC(C=C)
CCC=CCOC(=O)NC5CC(C)(C)CC(C)(CNC(=O)OC=CCC(C=C)CCC=CCOC=C
CC(C=C)CCC=CCOC(=O)NC6CC(C)(C)CC(C)(CNC(=O)NC7CCCC(NC(=O)
NC8CC(C)(C)CC(C)(CNC(=O)Nc9cccc(NC(=O)NC%10CC(C)(C)CC(C)(
CN=C=O)C%10)n9)C8)n7)C6)C5)C4)n3)C2)C1
```

Translation Results:

Component types: IPDI, Poly bd, DAPy

Symbolic hypergraph string: $\mathcal{SHSHS}$

Production rules: $\{p_2, p_{12}, p_{10}, p_{13}, p_{12}, p_{10}, p_{13}, p_{13}, p_8, p_{14}\}$

9. Input SMILES:

C=CC(CC=COCC=CCCC(C=C)CC=COC(=O)Nc1ccc(Cc2ccc(NC(=O)OCCOC
COC(=O)Nc3ccc(Cc4ccc(NC(=O)OCC=CCCC(C=C)CC=COC(=O)Nc5ccc(
Cc6ccc(NC(=O)OCC=CCCC(C=C)CC=COC(=O)Nc7ccc(Cc8ccc(NC(=O)O
CC=CCCC(C=C)CC=COC(=O)Nc9ccc(Cc%10ccc(NC(=O)OCCOCCOC(=O)N
c%11ccc(Cc%12ccc(NC(=O)OCC=CCCC(C=C)CC=COC(=O)Nc%13ccc(Cc
%14ccc(NC(=O)OCC=CCCC(C=C)CC=COC(=O)Nc%15ccc(Cc%16ccc(N=C
=O)cc%16)cc%15)cc%14)cc%13)cc%12)cc%11)cc%10)cc9)cc8)cc7)
cc6)cc5)cc4)cc3)cc2)cc1)CCC=CCOC=CCC(C=C)CCC=CCOC(=O)Nc1c
cc(Cc2ccc(N=C=O)cc2)cc1

Translation Results:

Component types: MDI, Poly bd, DEG

Symbolic hypergraph string: $\mathcal{HSHSHHSHSHSHHSH}$

Production rules: $\{p_2, p_6, p_{12}, p_3, p_4, p_6, p_4, p_6, p_4, p_6, p_3, p_4, p_6, p_4, p_6, p_5, p_{11}\}$

10. Input SMILES:

O=C(O)CCCCC(=O)OCCOC(=O)Nc1cccc2c(NC(=O)OC(=O)CCCCC(=O)OC
COC(=O)Nc3cccc4c(NC(=O)OC(=O)CCCCC(=O)OCCOC(=O)CCCCC(=O)O
CCOC(=O)Nc5cccc6c(NC(=O)Nc7cccc(NC(=O)Nc8cccc9c(NC(=O)Nc%
10cccc(NC(=O)Nc%11cccc%12c(NC(=O)Nc%13cccc(NC(=O)Nc%14ccc
c%15c(NC(=O)Nc%16cccc(NC(=O)Nc%17cccc%18c(NC(=O)Nc%19cccc
(NC(=O)Nc%20cccc%21c(NC(=O)OC(=O)CCCCC(=O)OCCOC(=O)Nc%22c
ccc%23c(NC(=O)OC(=O)CCCCC(=O)OCCOC(=O)CCCCC(=O)OCCOC(=O)C
CCCC(=O)OCCO)cccc%22%23)cccc%20%21)n%19)cccc%17%18)n%16)c
ccc%14%15)n%13)cccc%11%12)n%10)cccc89)n7)cccc56)cccc34)cc
cc12

Translation Results:

Component types: NDI, PEA, DAPy

Symbolic hypergraph string: $\mathcal{SHSHSHS}$

Production rules: $\{p_2, p_{12}, p_{10}, p_{12}, p_{10}, p_{13}, p_{13}, p_{13}, p_{13}, p_{13}, p_{12}, p_{10}, p_8, p_{14}\}$

S4. Generalized PolyGrammar to Other Polymers and Stereochemistry

The extended PolyGrammar for different types of copolymers and functional groups is illustrated as follows,

- for block copolymers,

$$
\begin{aligned}
&p_1 : None < \mathcal{X} > None: None \rightarrow h\mathcal{H}(0.5L_H)h \\
&p_2 : None < \mathcal{X} > None: None \rightarrow s\mathcal{S}(0.5L_S)s \\
&p_3 : None\ < h > \mathcal{H}(x): x \geq 1 \rightarrow h\mathcal{H}(x-1) \\
&p_4 : None\ < h > \mathcal{H}(x): x < 1 \rightarrow s\mathcal{S}(L_S) \\
&p_5 : None\ < h > \mathcal{H}(x): x < 1 \rightarrow Null \\
&p_6 : None\ < s > \ \mathcal{S}(x): x \geq 1 \rightarrow s\mathcal{S}(x-1) \\
&p_7 : None\ < s > \ \mathcal{S}(x): x < 1 \rightarrow h\mathcal{H}(L_H) \\
&p_8 : None\ < s > \ \mathcal{S}(x): x < 1 \rightarrow Null \\
&p_9 : \ \mathcal{H}(x) < h > None: x \geq 1 \rightarrow \mathcal{H}(x-1)h \\
&p_{10}: \mathcal{H}(x) < h > None: x < 1 \rightarrow \mathcal{S}(L_S)s \\
&p_{11}: \mathcal{H}(x) < h > None: x < 1 \rightarrow Null \\
&p_{12}: \ \mathcal{S}(x)\ < s > None: x \geq 1 \rightarrow \mathcal{S}(x-1)s \\
&p_{13}: \ \mathcal{S}(x)\ < s > None: x < 1 \rightarrow \mathcal{H}(L_H)h \\
&p_{14}: \ \mathcal{S}(x)\ < s > None: x < 1 \rightarrow Null
\end{aligned}
$$

- for alternating copolymers,

$$
\begin{aligned}
&p_1 : \ None < \mathcal{X} > None: None \rightarrow h\mathcal{H}(L)h \\
&p_2 : \ None < \mathcal{X} > None: None \rightarrow s\mathcal{S}(L)s \\
&p_3 : \ None\ < h > \mathcal{H}(x): x \geq 1 \rightarrow s\mathcal{S}(x-1) \\
&p_4 : \ None\ < h > \mathcal{H}(x): x < 1 \rightarrow Null \\
&p_5 : \ None\ < s > \ \mathcal{S}(x): x \geq 1 \rightarrow h\mathcal{H}(x-1) \\
&p_6 : \ None\ < s > \ \mathcal{S}(x): x < 1 \rightarrow Null \\
&p_7 : \ \mathcal{H}(x)\ < h > None: x \geq 1 \rightarrow \mathcal{S}(x-1)s \\
&p_8 : \ \mathcal{H}(x)\ < h > None: x < 1 \rightarrow Null
\end{aligned}
$$

$$p_9: \ \mathcal{S}(x) \ < s > None: x \geq 1 \rightarrow \mathcal{H}(x-1)h$$
$$p_{10}: \ \mathcal{S}(x) \ < s > None: x < 1 \rightarrow Null$$

- for functional groups of polyacrylates,

$$p_1: \ b(x) < \mathcal{F} > None: y = x + z \rightarrow c(y)\mathcal{A}(z)$$
$$p_2: \ None < \mathcal{A}(z) > None: z > 1 \rightarrow [\mathcal{A}(z-1)]\mathcal{A}(1)$$
$$p_3: \ None < \mathcal{A}(z) > None: z \geq 1 \rightarrow [b(x)]\mathcal{F}$$
$$p_4: \ None < \mathcal{A}(z) > None: z \leq 1 \rightarrow None$$

To represent stereochemistry, we introduce the binary parameter "t" as the orientation indicator. $t = 0$ and $t = 1$ distinguish the two different orientations. Here we use polypropylene as an example. The symbol "$\mathcal{H}$" represents the monomer propylene. The first parameter x of $\mathcal{H}$ controls the chain length and the second one t controls the tacticity. For isotactic polypropylenes, the parameter t remains the same at each production. For syndiotactic polypropylenes, the parameter t is altered by performing $1 - t$. For atactic ones, the parameter can either be maintained or altered, so there are two possible production rules to expand the symbol along each direction. During the generation process, the rules are randomly selected and deployed as illustrated in Section 3.1.1. Detailed rules are shown as follows,

- for isotactic polypropylenes,

$$p_1: \ None < \mathcal{X} > \ None \ : \ None \rightarrow h\mathcal{H}(L, 0)h$$
$$p_2: \ None \ < h > \mathcal{H}(x,t): x \geq 1 \rightarrow h\mathcal{H}(x-1, t)$$
$$p_3: \mathcal{H}(x,t) < h > \ None: x \geq 1 \rightarrow \mathcal{H}(x-1, t)h$$
$$p_4: \mathcal{H}(x,t) < h > \ None: x < 1 \rightarrow Null$$
$$p_5: \ None < h > \mathcal{H}(x,t): x < 1 \rightarrow Null$$

- for syndiotactic polypropylenes,

$$p_1: \ None < \mathcal{X} > \ None \ : \ None \rightarrow h\mathcal{H}(L, 0)h$$
$$p_2: \ None \ < h > \mathcal{H}(x,t): x \geq 1 \rightarrow h\mathcal{H}(x-1, 1-t)$$
$$p_3: \mathcal{H}(x,t) < h > \ None: x \geq 1 \rightarrow \mathcal{H}(x-1, 1- \ t)h$$
$$p_4: \mathcal{H}(x,t) < h > \ None: x < 1 \rightarrow Null$$
$$p_5: \ None < h > \mathcal{H}(x,t): x < 1 \rightarrow Null$$

- for atactic polypropylenes,

$$p_1 : \ None < \mathcal{X} > \ None : \ None \rightarrow h\mathcal{H}(L, 0)h$$
$$p_2 : \ None \ < h > \mathcal{H}(x,t) \colon x \geq 1 \rightarrow h\mathcal{H}(x-1, t)$$
$$p_3 : \ None \ < h > \mathcal{H}(x,t) \colon x \geq 1 \rightarrow h\mathcal{H}(x-1, 1-t)$$
$$p_4 : \mathcal{H}(x,t) < h > \ None \colon x \geq 1 \rightarrow \mathcal{H}(x-1, t)h$$
$$p_5 : \mathcal{H}(x,t) < h > \ None \colon x \geq 1 \rightarrow \mathcal{H}(x-1, 1- \ t)h$$
$$p_6 : \mathcal{H}(x,t) < h > \ None \colon x < 1 \rightarrow Null$$
$$p_7 : \ None < h > \mathcal{H}(x,t) \colon x < 1 \rightarrow Null$$

Index	Production Rules	Generated Hypergraph Symbolic String
1	$\{p_1, p_2, p_3, p_2, p_3, p_2, p_3, p_5, p_4\}$	$\mathcal{H}(t=0)\mathcal{H}(t=0)\mathcal{H}(t=0)\mathcal{H}(t=0)\mathcal{H}(t=0)\mathcal{H}(t=0)\mathcal{H}(t=0)$
	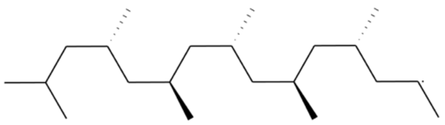	
2	$\{p_1, p_2, p_3, p_2, p_3, p_2, p_3, p_3, p_5, p_4\}$	$\mathcal{H}(t=0)\mathcal{H}(t=1)\mathcal{H}(t=0)\mathcal{H}(t=1)\mathcal{H}(t=0)\mathcal{H}(t=1)\mathcal{H}(t=0)\mathcal{H}(t=1)$
	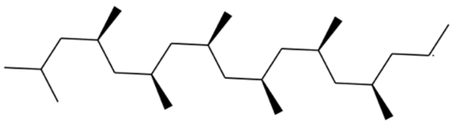	
3	$\{p_1, p_4, p_3, p_4, p_2, p_4, p_3, p_5, p_2, p_4, p_6, p_7\}$	$\mathcal{H}(t=0)\mathcal{H}(t=0)\mathcal{H}(t=1)\mathcal{H}(t=1)\mathcal{H}(t=0)\mathcal{H}(t=0)\mathcal{H}(t=0)\mathcal{H}(t=0)\mathcal{H}(t=1)\mathcal{H}(t=1)$
	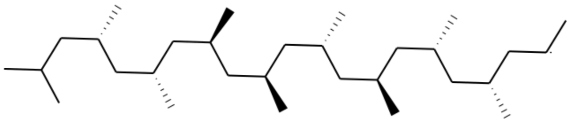	

Generated examples for these three types of polypropylenes, their corresponding hypergraph symbolic representations together with sequences of production rules are shown above, where the first, second, and third generated chain corresponds to isotactic, syndiotactic, and atactic polypropylenes, respectively.

S5. Extensions for Meta-ring structures

The production rules for the meta-ring structures are illustrated as follows,

$$p_1: \ None < \mathcal{X} > None \rightarrow h\mathcal{H}r\mathcal{H}h$$
$$p_2: \ None < h > \ \mathcal{H} \ \rightarrow h\mathcal{H}$$
$$p_3: \ \mathcal{H} \ < h > None \ \rightarrow \mathcal{H}h$$
$$p_4: None \ < h > \ \mathcal{H} \ \rightarrow h\mathcal{H}r$$
$$p_5: \ \mathcal{H} \ < h > None \rightarrow r\mathcal{H}h$$
$$p_6: \ \mathcal{H} \ < r > \ \mathcal{H} \ \rightarrow$$

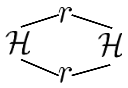

$$p_7: \ \mathcal{H} \ < r > \ \mathcal{H} \ \rightarrow r\mathcal{H}$$
$$p_8: \ \mathcal{H} \ < r > \ \mathcal{H} \ \rightarrow \mathcal{H}r$$
$$p_9: \ None < h > \ \mathcal{H} \ \rightarrow Null$$
$$p_{10}: \ \mathcal{H} \ < h > None \rightarrow Null$$

For simplicity, here we only show the context-sensitive grammar without parameters and only consider one type of molecular fragment. Note that all the proposed schemes involving parameters in this paper can be encoded in this grammar, the same for the setting of more fragment types. Compared to the original grammar, the major difference is production rule p_6, which has a graph instead of a string at right-hand side. This enables meta-ring structures in the production process. An example of the whole production process for a polymer network formed by cross-linking poly-(4-vinyl pyridine) (P4VP) with bis-Pd (II) complexes is illustrated in Figure S2.

Starting with the initial symbol "$\mathcal{X}$", we first get a string with the symbol "r". This "r" symbol can be regarded as an indicator of the ring position which will be further expand-

ed to a complete meta-ring. Then, the production rule p_6 achieves the expansion. As shown in the figure, the symbolic string now, or more concisely the symbolic graph, now has a circle inside. The rule p_6 can be adopted multiple times to enable multiple rings in the symbolic graph as the following production sequences show. After all the non-terminal symbols are removed by p_9 and p_{10}, the symbolic graph can be reformatted to an isomorphic form, which is more obvious to be recognized as a polymer network with interactions between chains. The "r" symbols can be either treated as hydrogen bonding or crosslinkers connecting chains. In this case, the symbol "r" represents the bis-Pd (II) complexes and the symbol "$\mathcal{H}$" represents the P4VP. This extension supplements our PolyGrammar with an important ability to handle meta-ring structures.

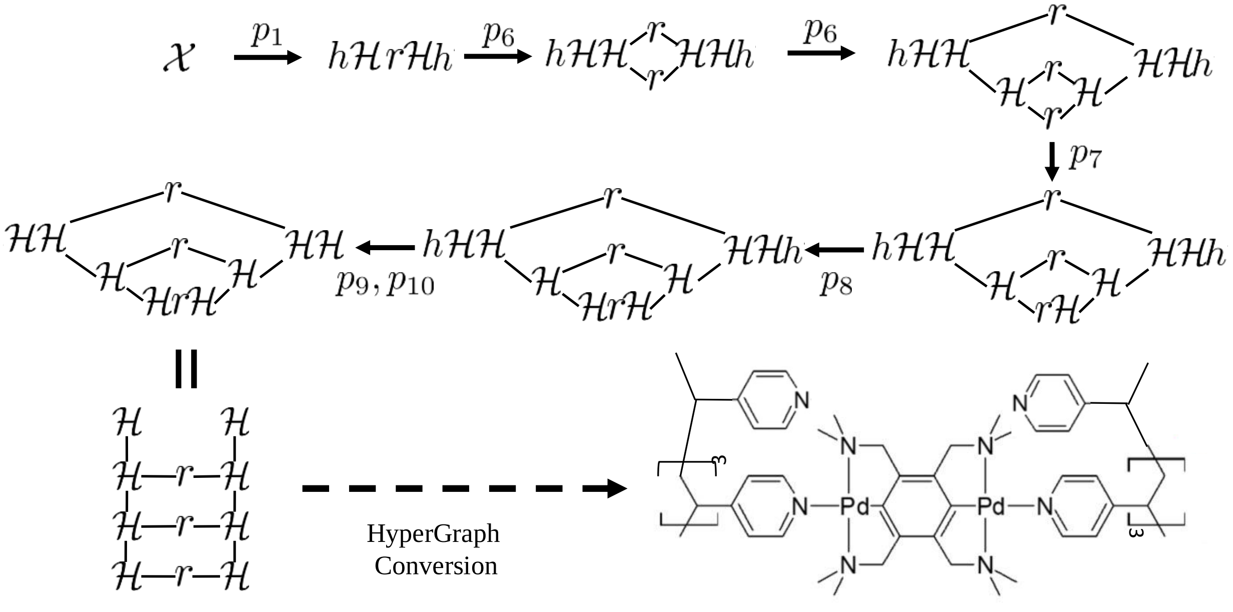

Figure S2. An illustrative example of PolyGrammar extension to meta-ring structures. The example shows the whole production process for a polymer network formed by cross-linking poly-(4-vinyl pyridine) (P4VP) with bis-Pd (II) complexes.

S6. Pseudo-code of Translation from SMILES

The inverse design process contains three parts: disconnecting carbamate bonds, constructing the hypergraph, and BFS searching for rules matching. Pseudo code is illustrated as follows.

Algorithm 1: Pseudo-code of translation from SMILES.

Input:

SMILES string of polyurethane chain $\mathcal{P}$

The set of production rules of PolyGrammar $\{p_i | \ i = 1, \ldots, N\}$

The set of SMILES strings of isocyanate candidates $\{\mathcal{J}_i | \ i = 1, \ldots, N_J\}$

The set of SMILES strings of macrodiol candidates $\{\mathcal{D}_i | \ i = 1, \ldots, N_D\}$

The set of SMILES strings of chain-extender candidates $\{\mathcal{C}_i | \ i = 1, \ldots, N_C\}$

Output:

The sequence of the production rules $\{p_k | k = i_i, \ldots, i_K\}$ to produce $\mathcal{P}$

```
Function GetSubstructMatches(a, s):
/*
input:
    SMILES string, a (the substructure sought)
    SMILES string, s (the total molecule to be searched)
output:
    an array of integers or ∅, P(positions in s at which a is found)
*/
/* standard substructure matching algorithm */
return P;

Function KMPMatching(a, s):
/*
input:
    SMILES string, a (the word sought)
    SMILES string, s (the text to be searched)
output:
    an array of integers, P positions in s at which a is found)
*/
/* standard KMP string matching algorithm */
return P;

Function ConstructGraph(a, n):
/*
input:
    adjacency matrix, a
    an array of nodes, n
output:
    an undirected graph, G
*/
/* standard undirected graph construction algorithm */
return G;

Function BFSearch(G):
/*
input:
```

```
    an undirected graph, G
  output:
    an array of traversed edges, e
  */
  /* standard Breadth-first Search algorithm */
  return e;

/* 1. Disconnecting carbamate bonds */
CBondSMILES ← SMILES of Carbamate Bond;
CBondIdx ← GetSubstructMatches(CBondSMILES,𝒫);
n ← length(CBondIdx);
FragmentSet ← ∅;
CBondSet ← ∅;
StartIdx ← 0;
for i ← 0 to n do
  if i == n then
    location ← i;
  else
    EndIdx ← CBondIdx[i];
    FragmentSet ← FragmentSet ∪ {𝒫[StartIdx: EndIdx]};
    BondEndIdx ← EndIdx + length(CBond);
    CBondSet ← CBondSet ∪ {𝒫[EndIdx: BondEndIdx]};
    StartIdx ← BondEndIdx;
/* 2. Identifying the symbol type and constructing the hypergraph */
n ← length(FragmentSet);
AdjacencyMatrix ← n × n zero matrix ;
HyperNodes ← ∅;
/* get the adjacency matrix */
for i ← 0 to n − 1 do
  CBond ← CBondSet[i];
  CBondStart ← CBond[0];
  CBondEnd ← CBond[end];
  for j ← 0 to n − 1 do
    Fragment ← FragmentSet[i];
    if CBondStart in Fragment then
      StartIdx = j;
    else if CBondEnd in Fragment then
      EndIdx = j;
  AdjacencyMatrix[StartIdx][EndIdx] = 1;
  AdjacencyMatrix[EndIdx][StartIdx] = 1;
/* get the symbol type */
for i ← 0 to n − 1 do
  if {KMPMatching(Fragment,ℐ_i)} is not ∅ then
    HyperNodes ← HyperNodes ∪ {'ℋ'};
  else if {KMPMatching(Fragment,𝒟_i)} is not ∅ then
    HyperNodes ← HyperNodes ∪ {'𝒮'};
  else if {KMPMatching(Fragment, 𝒞_i)} is not ∅ then
    HyperNodes ← HyperNodes ∪ {'u'};
HyperGraph ← ConstructGraph(AdjacencyMatrix, HyperNodes);
/* 3. BFS searching the graph and rules matching */
```

$EdgeList \leftarrow$ `BFSearch(`$HyperGraph$`);`

$\mathcal{P} \leftarrow \emptyset;$

$n \leftarrow$ `length(`$EdgeList$`);`

for $i \leftarrow 0\ to\ n-1$ do

  $Edge \leftarrow EdgeList[i];$

  $Pre \leftarrow Edge.predecessor;$

  $Suc \leftarrow Edge.successor;$

  $Rule \leftarrow \{p_i\}$`.find(`$<Pre, Suc>$`);`

  $\mathcal{P} \leftarrow \mathcal{P} \cup Rule;$

return $\mathcal{P}$

S7. Examples of Generated Polyurethane Chains

**Index**	**Diisocyanate**	**Macrodiol**	**Chain Extender**	**Generated Hypergraph Symbolic String**
1	DBDI	PTMO	DAPO	$\mathcal{HSHHSHHHHSHHHHHHH}$
	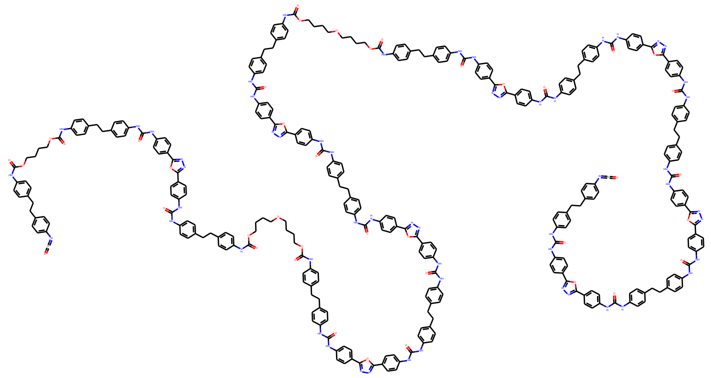			
2	TMDI	PCL	DAB	$\mathcal{HSHHSHHSHSHHHHS}$
	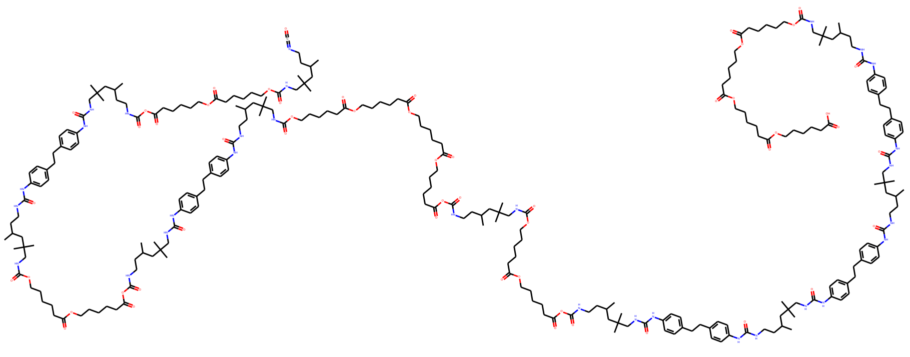			
3	NDI	PEA	DAPy	$\mathcal{SHSHHSHHS}$
	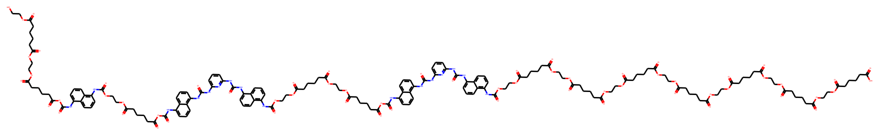			
4	DBDI	PCL	MDA	$\mathcal{HHSHSHHSHHSHSHS}$

	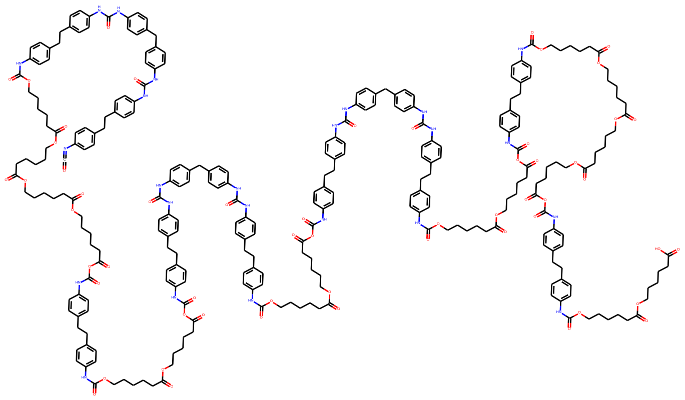			
5	MDI	PHA	BDO	$\mathcal{SHSHHHSHS}$
	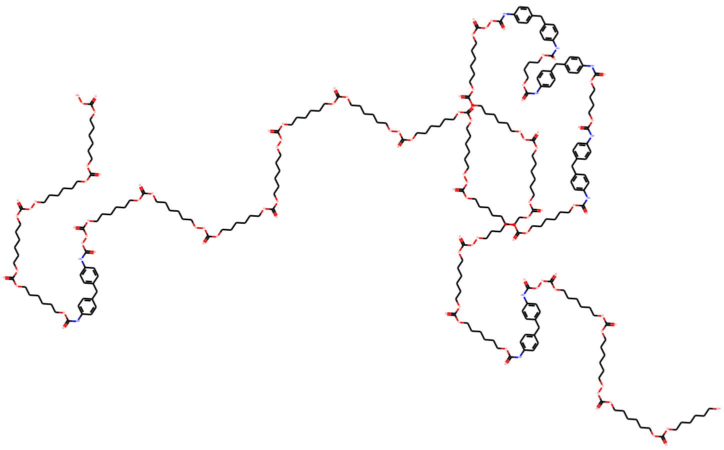			
6	DBDI	Poly bd	DAB	$\mathcal{SHSHHHSHHHSHHSH}$
	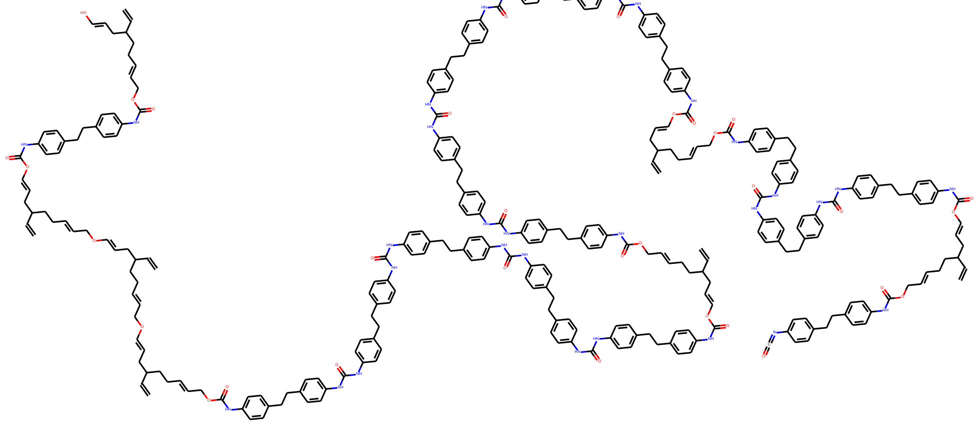			
7	NDI	PTMO	DAB	$\mathcal{SHHHHSHSHSHSHHS}$

	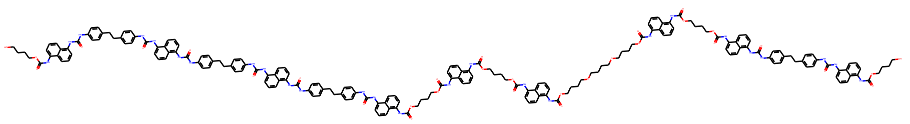			
8	MDI	PTMO	EG	$\mathcal{SHHSHSHSHSHS}$
	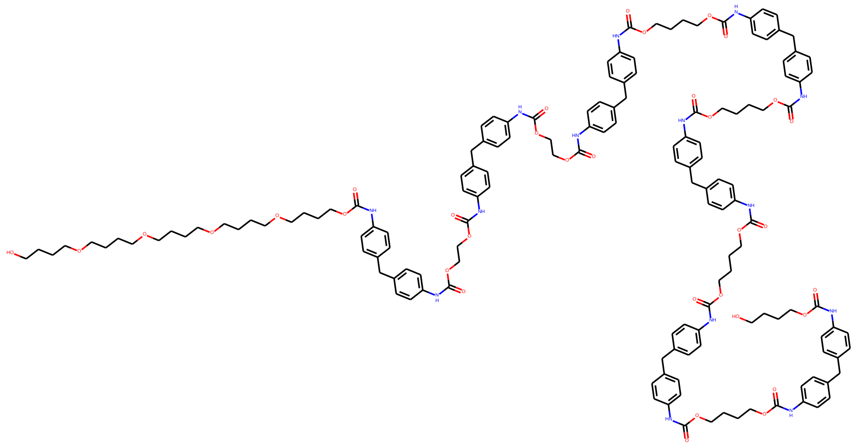			
9	HDI	PBU	DAPy	$\mathcal{HSHSHHSHSHHHSHS}$
	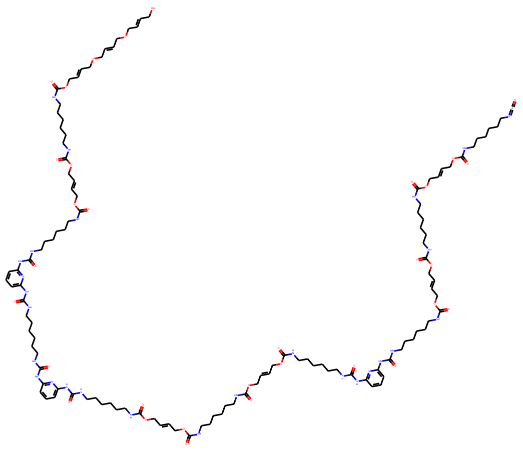			
10	MDI	PCL	DAPy	$\mathcal{SHHSHHSHHSHHHSH}$

	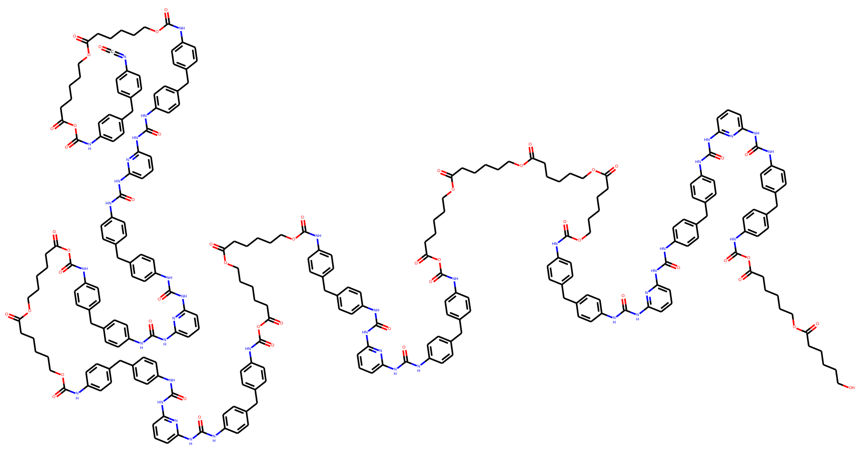

S8. Examples of Branched Polyurethane Chains

Index	Diisocyanate	Macrodiol	Chain Extender	Generated Hypergraph Symbolic String
1	TDI	PET	DAPy, 3THA	$\mathcal{HSHHHHHH}[\mathcal{HHHH}]\mathcal{SHHH}$
	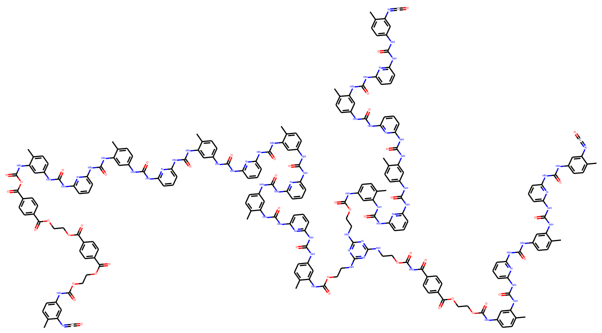			
2	DBDI	PTMO	MDA, 3THA	$\mathcal{HSH}[\mathcal{SH}]\mathcal{HSHSHH}[\mathcal{H}[\mathcal{S}]\mathcal{H}[\mathcal{S}]\mathcal{H}]\mathcal{HH}[\mathcal{S}]\mathcal{S}$
	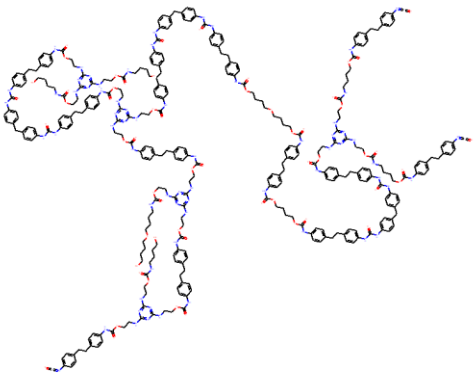			
3	DBDI	PCD	EG, 3THA	$\mathcal{HH}[\mathcal{H}]\mathcal{HHSHSHHSH}$

	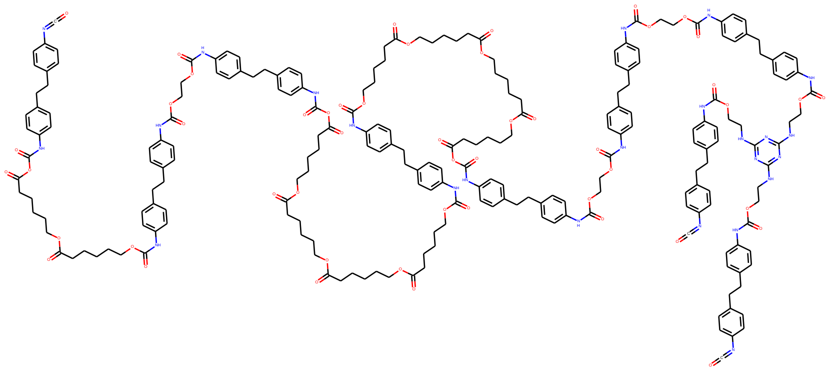			
4	HDI	PBA	BG, 3THA	$\mathcal{HSHSHH}[\mathcal{HS}]\mathcal{SHHS}$
	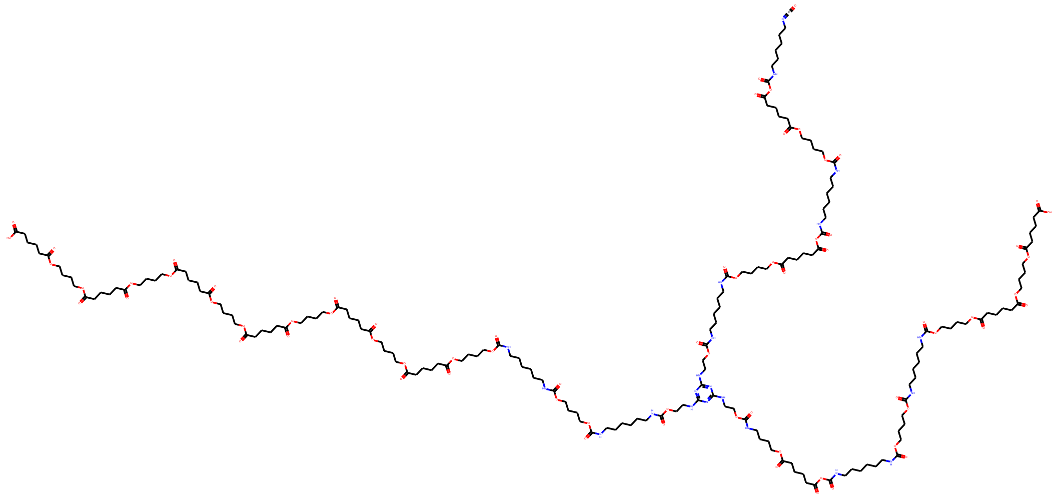			
5	NDI	CHDM	EG, 3THA	$\mathcal{HSH}[\mathcal{S}]\mathcal{H}[\mathcal{HS}]\mathcal{HHS}$
	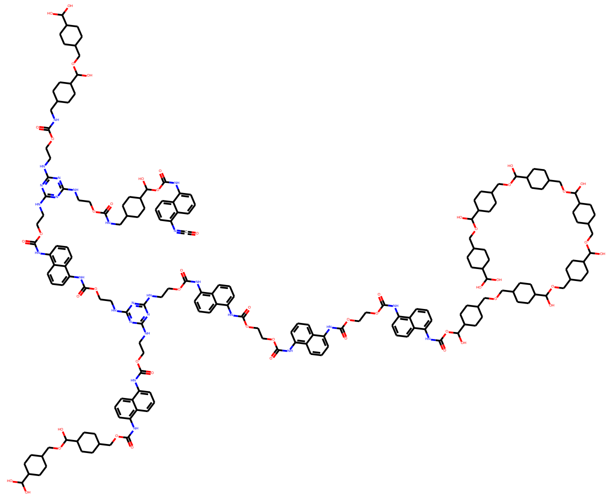			

S9. Examples of Acrylate's Functional Groups

Index	Generated Hypergraph Symbolic String	Acrylate's Functional Group
1	b(1)c(4)[b(1)c(1)][b(1)c(1)]b(1)c(4)[b(1)c(1)][b(1)c(1)]b(1)c(2)	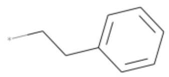
2	b(1)c(4)[b(1)c(1)][b(1)c(1)]b(1)c(4)[b(1)c(1)][b(1)c(1)]b(1)c(4)[b(1)c(1)][b(1)c(1)]b(1)c(4)[b(1)c(1)][b(1)c(1)]b(1)c(4)[b(1)c(1)][b(1)c(1)]b(1)c(4)[b(1)c(1)][b(1)c(1)]b(1)c(4)[b(1)c(1)][b(1)c(1)]b(1)c(4)[b(1)c(1)][b(1)c(1)]b(1)c(1)	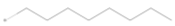
3	b(1)c(4)[b(1)c(4)[b(1)c(1)][b(1)c(1)]b(1)c(1)][b(1)c(1)]b(1)c(4)[b(1)c(1)][b(1)c(1)]b(1)c(4)[b(1)c(1)][b(1)c(1)]b(1)c(1)	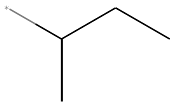
4	b(1)c(4)[b(1)c(4)[b(1)c(1)][b(1)c(1)]b(1)c(1)][b(1)c(4)[b(1)c(1)][b(1)c(1)]b(1)c(1)][b(1)c(4)[b(1)c(1)][b(1)c(1)]b(1)c(1)]	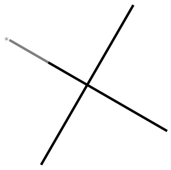
5	b(1)c(4)[b(1)c(1)][b(1)c(1)]b(1)c(4)[b(1)c(1)][b(1)c(1)]b(1)c(2)b(1)c(4)b(1)c(1)[b(1)c(1)][b(1)c(1)]	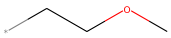
6	b(1)c(2)b(1)c(4)[b(1)c(1)][b(1)c(1)]b(1)c(2)b(1)c(4)[b(1)c(1)][b(1)c(1)]b(1)c(1)	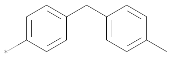
7	b(1)c(4)[b(2)c(2)]b(1)c(3)[b(1)c(1)]b(1)c(1)	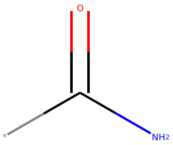
8	b(1)c(2)b(2)c(2)b(1)c(4)[b(1)c(1)][b(1)c(4)[b(1)c(1)][b(1)c(1)]b(1)c(1)b(1)c(4)[b(1)c(1)][b(1)c(1)]b(1)c(1)	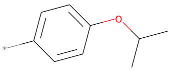
9	b(1)c(4)[b(1)c(2)b(1)c(1)][b(1)c(1)]b(1)c(4)[b(1)c(4)[b(1)c(1)][b(1)c(1)]b(1)c(1)][b(1)c(4)[b(1)c(1)][b(1)c(1)]b(1)c(1)][b(1)c(4)[b(1)c(1)][b(1)c(1)]b(1)c(1)]	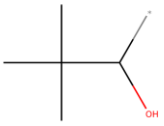

10	$b(1)c(2)b(1)c(2)b(1)c(4)[b(1)c(4)[b(1)c(1)][b(1)c(1)]b(1)c(1)]$ $[b(1)c(4)[b(1)c(1)][b(1)c(1)]b(1)c(1)][b(1)c(4)[b(1)c(1)][b(1)c(1)]$ $b(1)c(1)]b(1)c(2)b(1)c(2)b(1)c(1)$	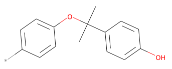

S10. Abbreviations and Acronyms

MDI	4,4’-methylenebis(phenyl isocyanate)
TDI	toluene-diisocyanate
DBDI	4,4’-dibenzyl diisocyanate
HDI	1,6-diisocyanatohexane
HMDI	hydrogenated MDI
IPDI	isophorone diisocyanate
NDI	1,5-Naphthalene diisocyanate
TMDI	2,2,4-trimethyl-1,6-hexamethylelne diisocyanate
PEG / PEO	poly(oxyethylene) glycol
PEA	poly(ethylene adipate)diol
PBA	poly(butane adipate) diol
PTMO / PTHF	poly(oxytetramethylene) diol / polytetrahydrofurane diol
PBU	poly(butadiene)diol
PCL / PCD	polycaprolactone diol
PHA	polyhexamethylene carbonate glycol
PET	polyethylene terephthalate
PLA	polylactic acid (lactic acid)
CHDM	1,4-cyclohexane dimethanol
Poly bd	polybutadiene diol
BD / BG / BDO	1,4-butanediol
EG	ethylene glycol
DEG	diethylene glycol
DAPO	2,5-bis-(4-amino-phenylene)-1,3,4-oxadiazole
DAB	4,4’-diamino-dibenzyl

DAPy	2,6-diamino-pyridine
MDA	4,4’-methylene-dianiline
